# Transcatheter Aortic Valve Implantation: All Transfemoral? Update on Peripheral Vascular Access and Closure

**DOI:** 10.3389/fcvm.2021.747583

**Published:** 2021-09-29

**Authors:** Nils Perrin, Guillaume Bonnet, Lionel Leroux, Réda Ibrahim, Thomas Modine, Walid Ben Ali

**Affiliations:** ^1^Structural Heart Intervention Program, Montreal Heart Institute, Montreal, QC, Canada; ^2^Cardiology Division, Geneva University Hospitals, Geneva, Switzerland; ^3^Service Médico-Chirurgical: Valvulopathies-Chirurgie Cardiaque-Cardiologie Interventionelle Structurelle, Hôpital Cardiologique de Haut Lévèque, CHU Bordeaux, Bordeaux, France

**Keywords:** transcatheter aortic valve implantation, vascular access, transfemoral, alternative access, vascular complication

## Abstract

Transfemoral access remains the most widely used peripheral vascular approach for transcatheter aortic valve implantation (TAVI). Despite technical improvement and reduction in delivery sheath diameters of all TAVI platforms, 10–20% of patients remain not eligible to transfemoral TAVI due to peripheral artery disease. In this review, we aim at presenting an update of recent data concerning transfemoral access and percutaneous closure devices. Moreover, we will review peripheral non-transfemoral alternative as well as caval-aortic accesses and discuss the important features to assess with pre-procedural imaging modalities before TAVI.

## Introduction

Transcatheter aortic valve implantation (TAVI) has become the new standard of care for patients suffering from symptomatic severe aortic stenosis at high or intermediate surgical risk and is considered as a reasonable alternative to surgery for low risk patients (1–6). Transfemoral access remains the most widely used peripheral vascular approach for TAVI. Current international guidelines recommend transfemoral access as the gold standard for TAVI (7, 8), with American guidelines suggesting even reconsidering surgery for patients in whom anatomy is not suitable for transfemoral access (8). However, concomitant severe peripheral artery disease is frequent in this population and increases the risk of vascular complications. In order to allow direct comparison in the literature, it is recommended to report vascular complications according to the latest Valve Academic Research Consortium (VARC)-3 criteria (9). Accordingly, vascular complications are separated in major or minor complications including closure device failure. Direct impact of major vascular complications on mortality following TAVI has been well reported using the preceding VARC-2 criteria (10, 11). Interestingly, the latest and recent VARC-3 criteria have introduced a separate section defining access-related non-vascular complications referring to surrounding non-vascular structure damage (9).

Despite technical improvement and reduction in delivery sheath diameters of all TAVI platforms, 10–20% of patients remain not eligible to transfemoral TAVI due to peripheral artery disease (12, 13). Accordingly, alternative routes include non-transfemoral peripheral (transsubclavian or transcarotid) or central (caval-aortic, transapical, and direct aortic) vascular approaches. Direct comparison of outcomes between transfemoral and alternative TAVI is difficult since these latter patients present usually more severe comorbidities and are considered at higher procedural risk.

In this review, we aim at presenting an update of recent data concerning transfemoral access and percutaneous closure devices. Moreover, we will review peripheral alternative as well as caval-aortic accesses and discuss the important features to assess with pre-procedural imaging modalities before TAVI. Transapical and direct aortic TAVI will not be addressed in this review since strong evidence has shown worse outcomes in comparison to other alternative accesses and are only rarely considered nowadays (14–16).

## Pre-procedural Planning

Pre-procedural planning is a key step for running a successful TAVI program. Among others, pre-procedural imaging allows both precise aortic root anatomy characterization and peripheral vessel assessment. Routine and systematic use of cardiac multislice computed tomography (MSCT) is currently strongly recommended before TAVI procedures (17). When considering vascular access, MSCT has been reported to predict vascular complications with greater predictive value than traditional peripheral angiography (18). Vascular minimal diameter, tortuosity, and extend and distribution of calcification are major predictors of vascular complications and directly impact feasibility of transfemoral TAVI (19, 20). More detailed specificities of peripheral vascular assessment will be discussed below for each access. Several softwares are nowadays available for MSCT imaging analysis and structure measurements. Among our favorites, FluoroCT is a lightweighted software designed by two interventional cardiologists. It allows operators to perform all the required measurement and analysis of structures before structural heart interventions. Although free, use of FluoroCT needs some learning skills as reconstruction (for example to obtain adequate aortic annulus alignment) should be performed by hand with no automatically reconstruction features. On the other hand, 3mensio (Pie Medical Imaging) offers separate modules addressing specifically each valve or peripheral vasculature with a very intuitive user interface and automatically detection of several structures simplifying procedural planning and measurements. These advanced characteristics are however available at an expensive price imposing costumers to by each module separately.

## Transfemoral Access

### Data

Recent data from the large Society of Thoracic Surgeons–American College of Cardiology Transcatheter Valve Therapy (STS-ACC TVT) registry (*n* = 276,316) reported an increasing proportion of patients undergoing transfemoral TAVI from 2013 to 2019, with 95% of all TAVI performed through a femoral access in 2019 (21). Interestingly, proportion of transfemoral procedures dropped from 76% in 2012 to 47% in 2013 as a consequence of alternative access site approval by the Food and Drug Administration (FDA). Since then, a constant increase in transfemoral procedure proportion was observed.

The PARTNER 2 study, who randomized intermediate risk patients to TAVI with a balloon expandable device or surgery, consolidated data regarding benefits of the transfemoral access by reporting superiority of TAVI in patients undergoing transfemoral TAVI in terms of mortality and disabling stroke (hazard ratio 0.79, *p* = 0.05, TAVI vs. surgery). This superiority was no longer true when considering all vascular approach TAVI procedures compared to surgery.

The national prospective French registry (FRANCE TAVI) recently contributed significantly to the topic by comparing data from 21,611 patients undergoing transfemoral (92.5%) and non-transfemoral peripheral vascular access TAVI (22). After performing a pre-specified propensity score-based matching for comparison of both groups, Beurtheret et al. reported similar procedural mortality (OR of 1.29; 95% CI: 0.87–1.94) and stroke rates (OR of 1.38; 95% CI: 0.88–2.19) between the transfemoral and non-transfemoral TAVI groups of patients, respectively (22). Interestingly, results remained similar when the analysis were performed on patients undergoing TAVI in the more recent half of study period and in intermediate-high/high volume centers (>105 procedures/year).

### Pre-procedural Imaging Specificities

MSCT is the imaging modality of choice for pre-procedural planning in all patients. While a minimal vessel lumen diameter from the left or right common femoral artery to the aortic valve of ≥5.5 mm is recommended with current 18 French delivery systems, the InLine Evolut R and Pro+ 23–29 mm valves (Medtronic) require a minimal diameter of ≥5 mm. Attention should be paid to perform the measures at the location of maximal stenosis and perpendicular to the long axis of the vessel. This recommended minimal lumen diameter cut-off considers a certain degree of vessel distention. Accordingly, calcification extending >270° of the vessel circumference at any level from the common femoral artery to the aorto-iliac bifurcation requires larger minimal lumen diameter in order to allow successful sheaths (Edwards system) or direct delivery system (Medtronic system) insertion. Moreover, calcifications located at the anterior part of the common femoral vessel should be identified as it may prevent percutaneous vessel puncture and percutaneous closure. Finally, significant tortuosity by itself is associated to vascular complications (23). However, in some cases without heavy ilio-femoral calcifications, tortuous vessels may be straighten using a stiff guidewire (24).

### Procedure

Downsizing of current prosthesis delivery catheters has allowed percutaneous transfemoral access to become the standard of care. Percutaneous transfemoral TAVI was widely rapidly used in clinical practice by most of TAVI centers even though no strong data comparing percutaneous to surgical cut-down exist. No randomized clinical trial comparing both techniques has ever been published. The largest and more recent data come from an Asian propensity score matching study comparing outcomes of patients undergoing transfemoral percutaneous or surgical cut-down TAVI using the Edwards Sapien XT valve. As part of the OCEAN TAVI registry, Kawashima et al. (25) reported among 586 patients (305 percutaneous, 281 surgical cut-down) significantly shorter procedural times, lower major vascular complication and bleeding rates, and shorter hospital length of stays in the percutaneous group. In the Brazilian TAVI registry including 402 patients, combined incidence of all-cause mortality, life-threatening bleedings and major vascular complications did not differ at 1 year between patients undergoing transfemoral percutaneous or surgical cut-down TAVI (26).

**Figure 1:**
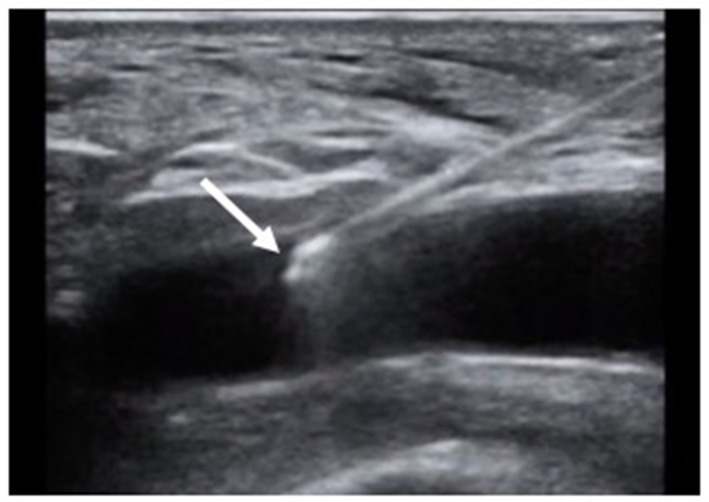
Doppler-guided puncture of the anterior wall of the common femoral artery. Tip of the needle inserted in the lumen of the artery (arrow).

Traditionally, the common femoral artery is punctured at the level of the center of the femoral head as localized by fluoroscopy. However, the bifurcation height varies in the general population. More recently, Doppler-guided femoral puncture (Figure 1) has been adopted to optimize femoral puncture and secure delivery sheath insertion. Doppler-guided puncture allows precise femoral bifurcation identification and highlights anterior wall calcifications. After identification of the femoral bifurcation in an axial view, the transducer is moved 1–2 centimeters more proximally. The common femoral artery is thereafter punctured by avoiding calcifications either in the axial view by keeping the needle almost vertical or by rotating the transducer 90° counterclockwise in order to obtain a longitudinal view of the artery while inserting the needle at 45° from the skin (Figure 1). While the axial view allows precise anterior arterial wall puncture, the longitudinal view gives the operator a better imaging of the needle entry in the artery. Even though widely used in clinical practice for several years, very few data assessing Doppler-guided femoral puncture outcomes have been reported. Only recently, Vincent et al. published the first propensity score matched comparison between Doppler- and fluoroscopic-guided femoral punctures for transfemoral TAVI (*n* = 95). Vascular and bleeding complications were largely reduced in the Doppler-guided group in comparison to the fluoroscopic-guided group (vascular complications 16.8 vs. 6.3%, *p* = 0.023 and life-threatening or major bleedings 22.1 vs. 6%, *p* = 0.04, respectively) (27). Moreover, micropuncture access set use (Cook Medical, Bloomington, IN) allows to confirm height of the puncture according to both the femoral head and femoral bifurcation (28). In case of inadequate puncture, the four French introducer is removed. Manual compression is performed before the new puncture is performed. Operators should however keep in mind that the 0.018 inch guidewire may easily perforate small arterial branches. We therefore recommend to novel operators to advance the guidewire under fluoroscopic guidance.

In case of heavy calcified ilio-femoral axis with borderline minimal vessel diameter, intravascular lithotripsy has been reported as feasible and safe for transfemoral TAVI. Indeed, intravascular lithotripsy disrupts intimal and medial calcification and allows large bore sheath insertion after increasing vessel compliance. The only reported experience yet relies on 42 successful TAVI patients with a target lesion diameter of 4.3 ± 1.1 mm, average stenosis of 58.6 ± 17.5% and average maximum calcium arc of 265.5 ± 88.3°. No access site perforation or dissection were reported (29).

### Pre-closure Devices

Commercially available CE mark vascular closure devices include the *Prostar XL* and *ProGlide* (Abbott Cardiovascular, suture-based), the *Manta* (Teleflex, collagen-based), the *PerQseal* (Vivasure Medical, patch-based) and the *InClosure* (InSeal Medical, membrane-based). Currently, the suture-based *ProGlide* and the collagen-based *Manta* are the most used vascular closure devices in TAVI and will be discussed in the present review. Both are inserted at the beginning of the TAVI procedure using a pre-closure technique.

**Figure 2:**
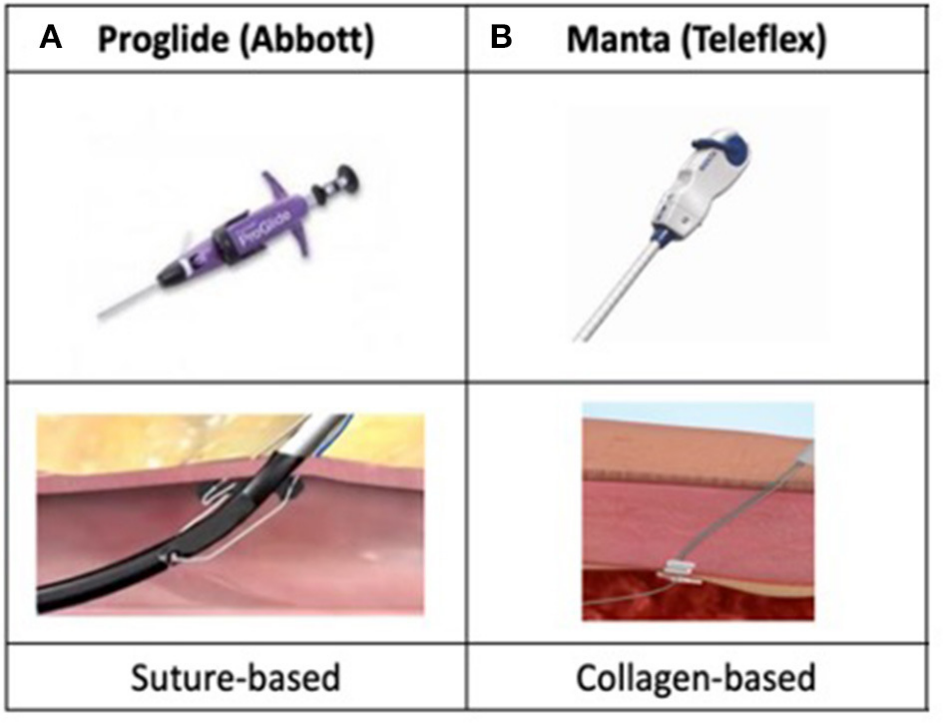
Percutaneous suture-based **(A)** and collagen-based **(B)** closure devices.

The *ProGlide* system (Figure 2A) closes the vessel by delivering a percutaneous suture at the level of the femoral arteriotomy. Typically, large TAVI delivery sheaths require the insertion of 2 devices. Pre-closure is successfully performed by inserting the devices at 10 and 2 o'clock position in the femoral artery. After removing the sheath at the end of the procure, the knocks are pushed and tightened against the vessel wall. Alternatively, some operators prefer to deploy 2 *ProGlide* in parallel (1 medial and 1 lateral) at 12 o'clock with the addition of a 6 or 8 French Angio-Seal (Abbott Vascular, collagen-based device designed for small accesses) at the end of the procedure.

Despite conflicting results among small reports comparing suture-based closure devices (*ProGlide* and *Prostar XL*), 2 recent large propensity score adjusted studies revealed superior efficacity and safety of the *ProGlide* closure device in terms of vascular and bleeding complications in TAVI patients (30, 31).

More recently, the *Manta* (Figure 2B), a collagen-based closure system for large percutaneous arteriotomies, has been developed with interesting features. It has been designed in 14 and 18 French allowing vessel closure after sheath removal as large as 25 French. An anchor is inserted in the artery before the bovine collagen plug is pushed against the vessel. Following the initial experience (*n* = 50) demonstrating safe closure of large arteriotomies (32), the American multicenter SAFE MANTA trial confirmed high technical success (98%) with a single device insertion in 99.6% of patients and low major vascular complications (4.2%) among 341 patients undergoing percutaneous transfemoral TAVI, endovascular abdominal aortic aneurysm repair, or thoracic endovascular aortic aneurysm repair (33). The *Manta* closure device has shown similar vascular complication rates with lower all-cause mortality and bleeding events when compared to the *ProGlide* system in a propensity-matched analysis of 111 matched pairs undergoing percutaneous transfemoral TAVI (34). Interestingly, a rapid learning curve of the *Manta* system use was observed since significant reduction in outcomes was not seen across the different period tertiles of the study.

## Transsubclavian/Axillary Access

### Data

Atherosclerosis tends to affect less the axillary and subclavian arteries in comparison to the ilio-femoral axis. Accordingly, transsubclavian or transaxillary (TS) approach has been one of the first peripheral alternative vascular access described for TAVI with progressive growing popularity. Data from the large STS-ACC TVT registry showed a progressive increase in TS access use through the years, reaching 2.5% of the TAVI procedures in 2019 (21). A further propensity matched analysis of the STS/ACC TVT registry reported a significant lower 30-day mortality (5.3 vs. 8.4%, *p* < 0.01) but higher stroke rate (6.3 vs. 3.1%, *p* < 0.05) with TS TAVI compared to traditional alternative accesses (transapical and transaortic), respectively (35). A slightly higher proportion of TS TAVI (3.2% of the patients) were performed in the FRANCE TAVI registry (*n* = 21,611). Among these latter patients, major vascular complications were reported in 1.3% of the patients and 4% of all TS approaches needed unplanned vascular repair (22). More recently, Van der Wulp et al. reported a vascular complication rate as high as 18.5% with however a very low major vascular complication rate of 0.5% (1 patient). Unplanned vascular repair was needed in 8.5% of the patients. Overall, procedural success was high (93.5% of the patients) (36). A small propensity-matched comparison between TF (*n* = 141) and TS (*n* = 141) access reported similar outcomes at 2 year in terms of procedural success (subclavian 98 vs. femoral 97%, *p* = ns), major vascular complications (5 vs. 8%, *p* = ns), life-threatening bleeding (8 vs. 6%, *p* = ns) and survival (74 vs. 74%, *p* = ns) (37). Finally, in a recent large meta-analysis including 79,426 patients undergoing TF vs. non-TF peripheral access TAVI (TS or TC), authors reported a trend toward a higher rate of vascular complication in the TS vs. TF group (RR, 1.30; 95% CI, 0.98–1.73), but this difference was no longer true when using more restricted adjusted data (38). Noteworthy, vascular complication definition did however not systematically rely on Valve Academic Research Consortium (VARC) criteria. Similarly, 30-day mortality was higher in the TS vs. TF group only when considering unadjusted data (RR, 1.54; 95% CI, 1.26–1.89). Stroke rates, however, were higher in patients undergoing TS TAVI compared to TF TAVI, using both adjusted and unadjusted data (unadjusted analysis: risk ratio (RR) of 2.28 [95% CI, 1.90–2.72]; adjusted analysis: odds ratio (OR) of 1.53 [95% CI, 1.05–2.22]).

### Pre-procedural Imaging Specificities

Similarly to TF pre-procedural planning, vessel minimal diameter, tortuosity, and calcification extension and localization are key elements to assess by MSCT. Subclavian artery take-off at the level of the aortic arch needs particular attention since it is a frequent localization of calcification. Significant subclavian artery to aortic arch angulation (>80°), as well as a horizontal aortic root may prevent successful valve delivery (39). A minimal diameter ≥5.5 mm is usually required for TS access in the absence of severe concentric calcifications. Interestingly, more than the minimal diameter by itself, Van der Wulp at al. (36) reported that the ratio of the sheath area to the axillary artery minimal diameter >1.63 is a strong independent predictor of vascular complication following TS TAVI. This ratio remains however experimental and needs further validation. Presence of patent internal mammal arteries in a patient with prior coronary artery bypass grafting is no longer considered as an absolute contraindication to TS approach even though alternative access are usually preferred.

### Procedure

**Figure 3:**
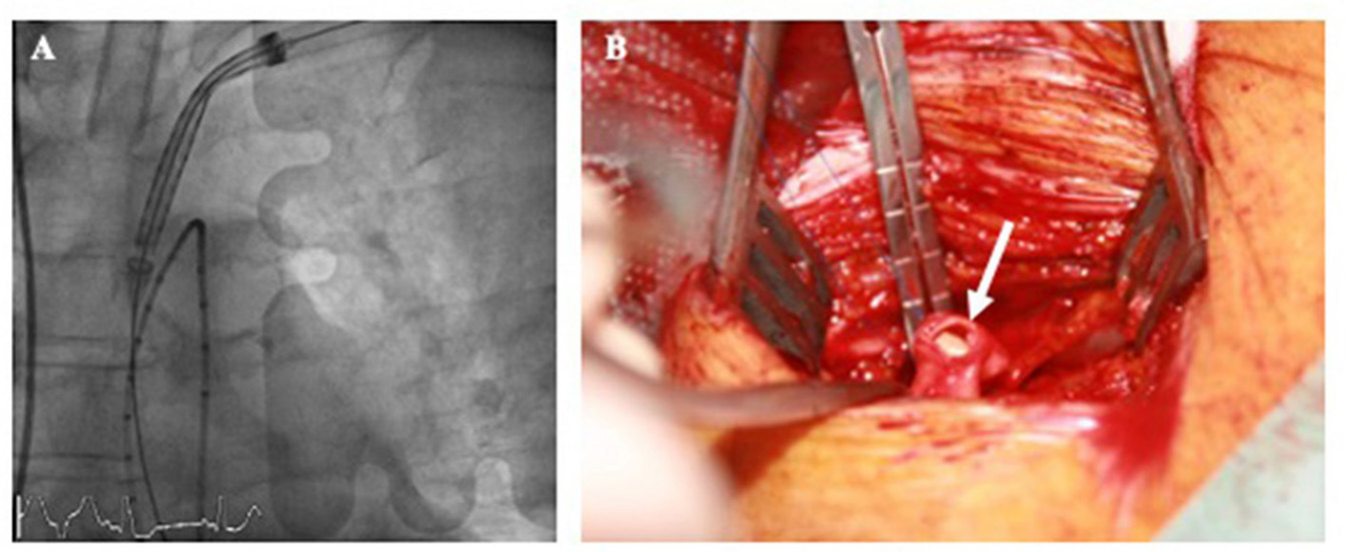
Left transsublavian access with valve delivery system insertion under fluoroscopic guidance at the level of the ascending aorta **(A)**. Subclavian arteriotomy after sheath removal (**B**, arrow).

It is worth to mention that the different histological structure of subclavian vessel wall makes it more prone to vascular complications with aggressive sheath insertion (mainly dissection or rupture). While the transfemoral artery is more muscular with a thicker and more fibrous adventia, the subclavian artery is characterized by a more thin and elastic wall (40). The left subclavian artery is usually preferred since the vascular path to the aortic root has less tortuosities and mimics TF access (Figure 3A). Until recently, most of TS accesses have been performed by surgical cut-down due to the fear of brachial plexus lesion with percutaneous puncture. A small 6–7 cm incision is performed 1 cm under the clavicle. Muscle fascia are dissected and particular attention should be paid to avoid neural structure injury. Once exposed, the subclavian artery is directly punctured. Subclavian arteriotomy (Figure 3B) is closed by surgical sutures at the end of the procedure. A vascular graft can be anastomosed end-to-side to the subclavian artery to allow safe valve delivery system insertion. In this case, the the graft is directly tied off at the end.

Fully percutaneous TS TAVI has been reported to be feasible and safe (41). Performing Doppler-guided punctures, Schafer at al. (42) reported a first German experience of 100 successive patients undergoing percutaneous TS TAVI with a 95% device success. No VARC-2 major vascular complication occurred. Interestingly, closure device failure occurred in 29.2% of the case, all while using the *ProStar* closure system. No closure device failure was reported when using the *ProGlide* system. More recently, the *Manta* system has shown favorable access site closure for TS TAVI (43, 44).

## Transcarotid Access

### Data

The first successful transcarotid (TC) TAVI has been described in 2010 (45). The most recent and largest data come from the French TAVI registry where 3.4% of patients underwent TC TAVI using the Edwards Sapien 3 prosthesis between 2014 and 2018 (46). Among 314 patients, procedural success was high (97%) with low 30-day mortality (3.2%) and cerebro-vascular ischemic event rate (1.6%). Major vascular complication or bleeding events were reported in 1.6 and 4.1% of the cases, respectively. Interestingly, when comparing TC (*n* = 911) to TS (*n* = 702) approach in the French TAVI registry, patients in the TC group had higher major bleeding rates (10 vs. 6.7%, *p* = 0.002, respectively) but lower major vascular complications rates (0.2 vs. 1.8%, *p* = 0.02, respectively). Stroke rate was similar between both groups (3.6 vs. 3.0%, *p* = 0.47) (22). TC TAVI has been compared to TS TAVI after propensity-matched scoring by Debry et al. in French multicenter registry. Interestingly, authors reported similar 30-day and 1-year mortality as well as 30-day stroke/transient ischemic attack (47). Surprisingly, minor bleeding (2.7 vs. 9.3%) and main access hematoma (3.6 vs. 10.3%) were significantly more frequent with the TC access.

### Pre-procedural Imaging Specificities

MSCT imaging of bilateral supraaortic pre-cerebral arteries is recommended as part of the pre-procedural planning. Ipsilateral calcification extension and localization as well as plaque at high risk of embolization should be assessed. Contralateral significant common carotid artery stenosis or occlusion usually contraindicates TC approach. A minimal lumen diameter of the common carotid artery ≥5.5 mm without >50% stenosis is required. Some centers recommend to evaluate the circle of Willis perfusion in order to assure adequate contralateral blood flow compensation during the TC TAVI procedure. However, no recommendation concerning routine pre-procedural circle of Willis assessment exist yet.

### Procedure

**Figure 4:**
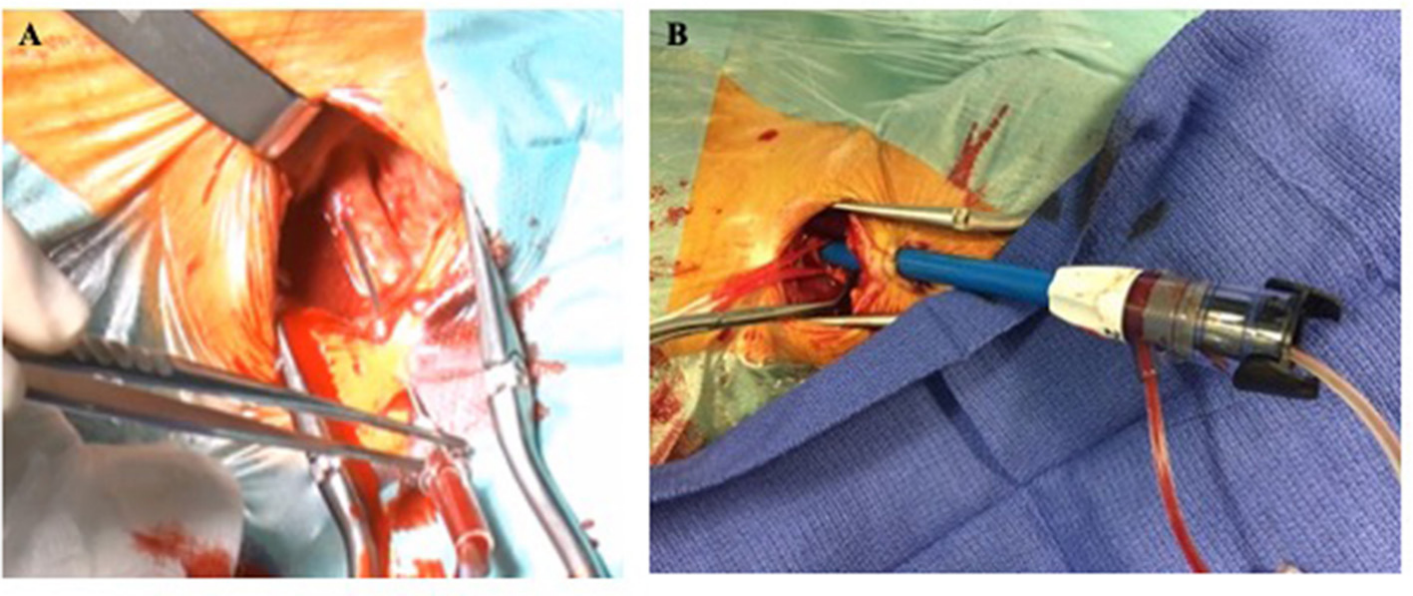
Left transcarotid surgical cut-down with anterior wall arterial puncture **(A)** and valve delivery sheath insertion **(B)**.

Left common carotid artery is usually preferred for similar aortic root alignment reasons than described in the TS section of this review. However, carotid vascular access should be performed where arterial disease is the worse in order to preserve arterial brain flow on the contralateral side during the TAVI procedure. TC access is systematically performed by surgical exposure of the common carotid artery and avoiding vagal nerve lesion. After anterior wall puncture (Figure 4A) and progressive arteriotomy dilatation, the delivery sheath (Edwards Certitude or Medtronic EnVeo R) is inserted (Figure 4B). A close follow-up of the regional cerebral oxygenation (rSO2) is useful and a >20% relative reduction from baseline (normal range = 55–78%) is a commonly adopted threshold that has been used in major randomized controlled trials of rSO_2_-guided interventions (44). Surgical carotid repair of the carotid artery is performed at the end of the procedure. Caution should be paid to avoid any air embolism before clamps are removed. Some centers use adjunctive perclose sutures in addition to surgical ligatures to guarantee adequate hemostasis. Interestingly, a French study analyzing data from 174 TC TAVI reported feasibility of a minimally invasive strategy using local anesthesia vs. general anesthesia. While 30-day and 1-year mortality was similar between both groups, patients undergoing general anesthesia suffered from more strokes (8.1 vs. 0%, *p* < 0.001, respectively) (48).

## Transcaval Access

### Data

Transcaval or caval-aortic TAVI access has been developed by Halabi at al. (49) and first reported in animals in 2013. Since then, Greenbaum at al. (50) described the first successful experience in humans in 2014. This technique remains however still experimental with limited data. Among 100 consecutive patients non eligible to transfemoral TAVI, percutaneous transcaval access was performed and successful in 99% of the cases. Access closure with a cardiac occluder was successful in all but 1 patient who required a covered stent implantation. Even though 30-day survival was good (92%), VARC-2 life-threatening bleeding and major vascular complication rates were high, respectively 7 and 13% (51). Interestingly, at 12 months, 93% of patients (77 of 83 patients) had CT-proven cavo-aortic fistula occlusion and only 1 patient had persistent asymptomatic patent fistula (52).

### Pre-procedural Imaging Specificities

MSCT is essential to identify the closest location for crossing from the inferior vena cava to the abdominal aorta. The crossing spot is chosen at the level of the infra-renal aorta by avoiding any significant aortic wall calcification or interfering abdominal structures (bowel loop). Derived fluoroscopic angles and landmarks according to lumber vertebrae are anticipated by MSCT imaging reconstruction.

### Procedure

Simultaneous aortography and venography are performed after positioning a single loop gooseneck snare in the abdominal aorta at the pre-identified crossing spot. A 6-French guiding catheter is inserted in the inferior vena cava and positioned toward the snare loop. The crossing apparatus, consisting of a microcatheter containing a 0.014 Inches stiff wire (for example Confienza Pro 12, Asahi) mounted in a 0.035-inches wire convertor, is advanced in the guiding catheter. Next, crossing from the inferior vena cava to the infra-abdominal aorta is performed using an electrosurgery ablation system connected to the extremity of the 0.014 Inches wire. Once caval-aorta communication is performed, the crossing system is exchanged for a stiff 0.035 Inches wire allowing the insertion of a 22–24-French introducer sheath in the aorta. Aortography assures adequate hemostasis before standard transfemoral TAVI procedure is performed. At the end of the procedure, caval-aorta communication is closed using occluder devices usually approved for patent ductus arteriosus or intracardiac defect closure (Amplatzer Duct Occluder or Amplatzer Muscular VSD occlude St. Jude Medical, St. Paul, Minnesota) (50). Retroperitoneal bleeding is definitively the most feared complication following transcaval access. However, the surrounding retroperitoneal space pressurizes while caval-aorta communication is performed. The highest retroperitoneal pressure in comparison to the venous pressure directs potential aortic bleeding preferentially in the venous system preventing most of the major or life-threatening bleedings.

## General Complication Prevention and Treatment

### Secondary Vascular Access

A second vascular access is required for angiographic guidance during prosthesis deployment. Influenced by data coming from coronary angiogram and percutaneous coronary intervention showing a significant reduction in vascular access-related complication using radial vs. femoral access (53), radial secondary access for TAVI has been progressively used. Encouraging results have been reported by a multicenter registry including 4,949 patients undergoing TAVI using as secondary access either a transfemoral or transradial access (81.1 and 18.9%, respectively) (54). VARC-2 defined secondary access-related complication rate was significantly higher in the transfemoral vs. transradial group with similar results after propensity score matching (4.7 vs. 0.9%, *p* < 0.001; major vascular complication, 1.8 vs. 0%, *p* < 0.001). Moreover, the transfemoral group suffered also from a higher 30-day stroke rate (3.1 vs. 1.6%, *p* = 0.043, respectively) and mortality (4.0 vs. 2.4%, *p* = 0.047, respectively) (54).

### Early Detection of Access-Related Bleedings

Undiagnosed vascular access-related bleedings may be dramatic since a significant blood loss usually occurs before patients become symptomatic or the bleeding is detected by an imaging modality. Based on tissue impedance change during bleedings, a new monitor has been developed (Early Bird Bleed Monitoring System, Saranas) to identify early subclinical periprocedural bleedings. Briefly, 2 separate electrodes attached on a 6 or 8 French introducer monitor bioimpedance change up to 12 h after the procedure. Visual and audible signals are provided by the system and categorized in three level of bleedings. The first-in-man study reported high level of agreement with MSCT (Cohen's kappa = 0.84) among 60 patients undergoing different endovascular procedure with large-bore vascular access (55). Although encouraging, the system still needs further investigations with larger data.

### Acute Management of Femoral Vascular Complications

At the time of femoral vascular closure, femoral crossover using a stiff wire may help prompt bleeding management by balloon occlusion (56). According to the severity of the bleeding or in case of flow limiting dissection, femoral covered self-expanding stent placement is safe and associated with favorable long-term outcomes (57). Crossover was traditionally performed through a contra-lateral femoral access. However, radial secondary access has been recently use to reduce access-related vascular complication rate (54). As a consequence, a modified crossover technique through the secondary radial access has been suggested with encouraging results (58, 59).

More over, heparin reversal has recently been reported to reduce significantly rate of major or life-threatening complications, without increasing the risk of thrombo-embolic events (including stroke or myocardial infarction) (60). However, results should be interpreted cautiously since heparin reversal was performed in presence of vascular complication at the beginning of the study whereas protamine was administered to all patients toward the end of the study.

## Conclusions and Future Perspectives

**Figure 5:**
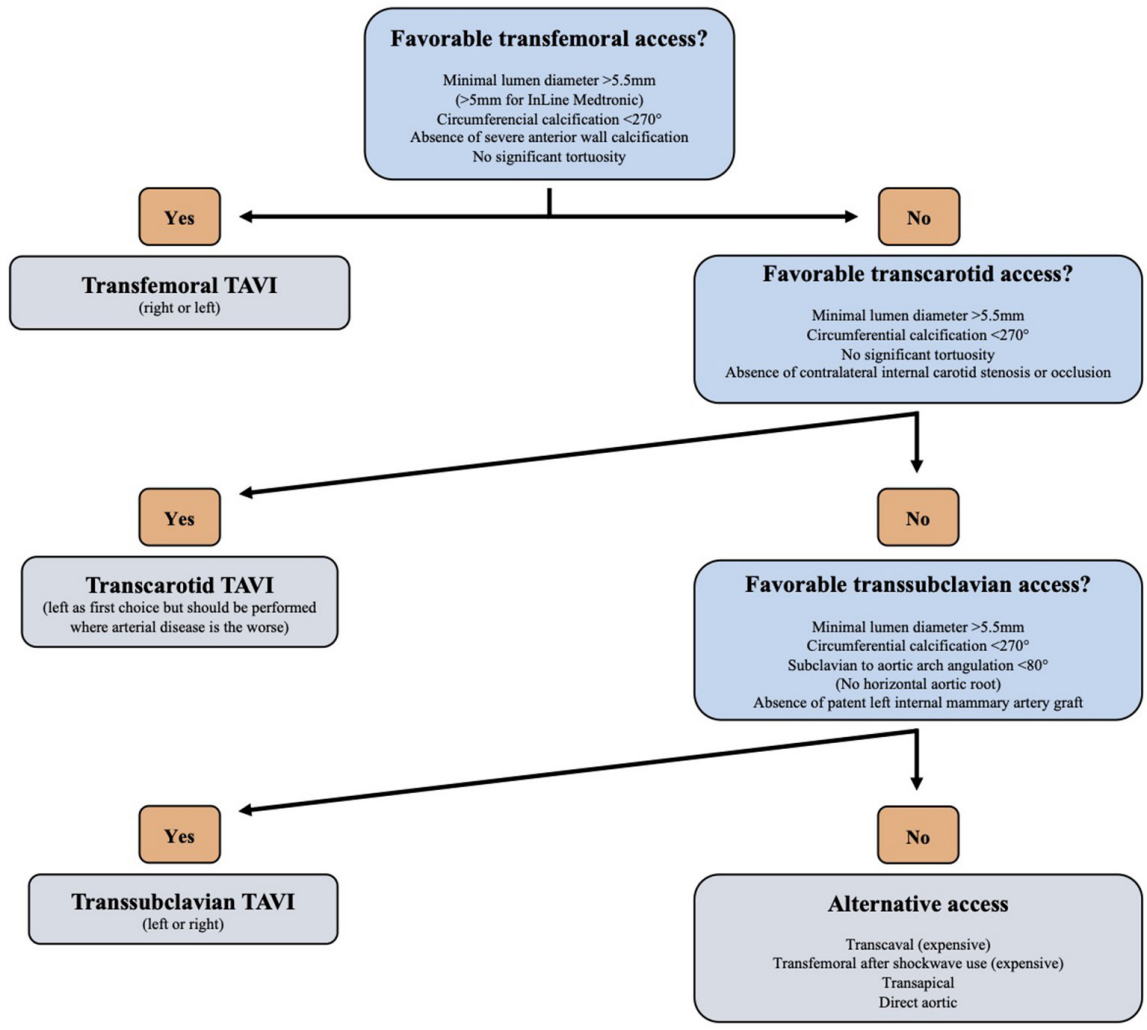
Vascular access decision algorithm.

In conclusion, percutaneous transfemoral TAVI remains the standard vascular approach. Pre-procedural imaging assessment of peripheral vascular disease plays a major role in the patient's comprehensive evaluation before a TAVI procedure. Figure 5 presents a decision algorithm for vascular access choice according to the authors' preference. Several alternative peripheral accesses have emerged over time with favorable outcomes. In particular, the TS and TC alternative accesses have been associated to similar outcomes to the TF TAVI in non-randomized but adjusted reports. The relative invasiveness of alternative access compared to TF TAVI have prevented the implementation of comparative studies. Moreover, direct comparison of TF and alternative access TAVI will remain limited by different risk profiles of the patients. Both, the TS and TC access are believed to become the new preferred alternative approaches in a near future. Percutaneous TS has been shown feasible and safe but needs further investigations and larger studies. Finally, caval-aortic TAVI is a reasonable approach when all peripheral accesses are precluded but is still experimental.

## Author Contributions

NP contributed to the design of the review and writing of the manuscript. GB, LL, RI, and TM revised the manuscript. WB contributed to the design of the review and reviewed critically the manuscript. All authors contributed to the article and approved the submitted version.

## Funding

NP has received research support from the Swiss National Science Foundation (P400PM_194483) and the Geneva University Hospitals.

## Conflict of Interest

The authors declare that the research was conducted in the absence of any commercial or financial relationships that could be construed as a potential conflict of interest.

## Publisher's Note

All claims expressed in this article are solely those of the authors and do not necessarily represent those of their affiliated organizations, or those of the publisher, the editors and the reviewers. Any product that may be evaluated in this article, or claim that may be made by its manufacturer, is not guaranteed or endorsed by the publisher.
